# MQ-MAC: A Multi-Constrained QoS-Aware Duty Cycle MAC for Heterogeneous Traffic in Wireless Sensor Networks

**DOI:** 10.3390/s101109771

**Published:** 2010-11-01

**Authors:** Muhammad Mostafa Monowar, Md. Obaidur Rahman, Choong Seon Hong, Sungwon Lee

**Affiliations:** Department of Computer Engineering, Kyung Hee University, Seocheon, Giheung, Yongin, South Korea

**Keywords:** multi-constrained QoS, heterogeneous traffic, duty cycle MAC

## Abstract

Energy conservation is one of the striking research issues now-a-days for power constrained wireless sensor networks (WSNs) and hence, several duty-cycle based MAC protocols have been devised for WSNs in the last few years. However, assimilation of diverse applications with different QoS requirements (*i.e.*, delay and reliability) within the same network also necessitates in devising a generic duty-cycle based MAC protocol that can achieve both the delay and reliability guarantee, termed as multi-constrained QoS, while preserving the energy efficiency. To address this, in this paper, we propose a Multi-constrained QoS-aware duty-cycle MAC for heterogeneous traffic in WSNs (MQ-MAC). MQ-MAC classifies the traffic based on their multi-constrained QoS demands. Through extensive simulation using ns-2 we evaluate the performance of MQ-MAC. MQ-MAC provides the desired delay and reliability guarantee according to the nature of the traffic classes as well as achieves energy efficiency.

## 1. Introduction

The enormous proliferation of micro-electro-mechanical systems (MEMS) [1], along with the wide adoption of wireless networking technologies have paved the way for large scale deployment of low-cost, low-power and multi-functional sensor networks. This also creates a great opportunity for the wide-spread utilization of various innovative applications in the foreseeable future. The distinct nature of these diverse applications also necessitates in designing effective communication protocols for WSNs.

Due to the power constraint of the battery-driven sensor nodes, energy conservation is given the utmost importance while designing the communication protocols for WSNs. As a consequence, several duty-cycle based MAC protocols [2,3] already exist in the literature, in which nodes periodically wake-up and sleep to avoid the idle listening, which is considered as the most dominant sources of energy consumption for the sensor nodes. These protocols can be broadly categorized as scheduled/synchronous and asynchronous, based on their wake-up schedule. Scheduled protocols [4–6] maintains synchronized wake-up schedule among neighbors. Asynchronous protocols [7–9] allow nodes to wake up independently of its neighbors, employing a preamble-based transmission approach. Focusing more on energy consumption, these protocols, however, overlooked the various quality-of-service (QoS) requirements of different applications. Meeting these requirements carries no less importance than providing energy efficiency, since if the requirements are not met properly, the objective of the application becomes futile, no matter how long the lifetime of the sensor network prevails.

Along with energy efficiency, the most important QoS metrics that different sensor network applications may require is the *delay* and *reliability*, together, which we denote as *multi-constrained QoS*. A sensor network may consist of multiple concurrent applications which have their respective QoS demands in terms of both delay and reliability. For instance, non-time critical applications has no strict delay and reliability requirements while the distributed control application requires both higher reliability and lower delay. The existence of such heterogeneous applications in the same network requires the treatment of each traffic based on their respective multi-constrained QoS requirements, for the preserving of application objective as well as for the proper utilization of the limited resources of WSNs.

Although offering the certain QoS along with providing duty cycling for energy efficiency has been given little attention so far, several proposals [10–12] fostered this issue. But these works considered only delay constraint as a QoS metric. Moreover, the main goal of these works is to reduce the latency as much as possible by controlling the duty-cycle and can not guarantee that the QoS requirements are actually met. Hence, a generic medium access control (MAC) solution is needed that provides energy efficiency along with the guarantee of multi-constrained QoS, which is demanded by the heterogeneous applications in WSNs and this type of solution, to best of our knowledge, remains un-addressed.

In this paper, we focus to design such a duty-cycle based MAC protocol, which faces the following challenges; (i) The duty-cycle based MAC protocols typically incur sleep delay which is one of the main hurdles to provide lower end-to-end delay for the delay intolerant traffic. (ii) Since nodes wake up periodically in maintaining duty-cycle and communicate among themselves during the active period, it results high contention and thereby collision which reduces the reliability and also increases the latency due to packet loss. (iii) Providing the same active period for all kinds of traffic inhibits in meeting the requirements of the traffic that possesses higher QoS requirements. (iv) Ensuring almost 100% reliability for loss intolerant applications necessitates a collision free transmission mechanism.

This paper introduces MQ-MAC, a novel multi-constrained QoS-aware duty-cycle MAC for heterogeneous traffic in wireless sensor networks which aims to address all the above mentioned challenges in satisfying the multi-constrained QoS demands along with providing duty-cycling for energy efficiency. Our solution includes a hybrid approach in wake-up scheduling (*i.e.*, both synchronous and asynchronous) to exploit the advantages of both the approaches in meeting the diverse QoS demands. We present traffic classification based on the multi-constrained QoS requirements of the heterogeneous applications. We provide distinct transmission periods for the traffic based on their classification to reduce the contention probability among diverse traffic classes. We also devise a novel reception-based TDMA scheduling during asynchronous approach, which assigns nodes reception schedule to ensure a delay efficient and collision free transmission. Furthermore, we develop analytical model of MQ-MAC and perform extensive simulations using ns-2 to evaluate the performance of MQ-MAC.

The remainder of the paper is organized as follows: Section 2 summarizes the related works. Some preliminaries behind our protocol design are stated in Section 3. The detailed design of MQ-MAC is presented in Section 4. Section 5 demonstrates the performance of our protocol, evaluated using ns2, and finally, in Section 6, we present our concluding remarks.

## 2. Related Work

In recent years, energy-efficient duty-cycle MAC protocols have been a very prominent research area for WSNs. S-MAC [4], T-MAC [6], D-MAC [13], R-MAC [5], DW-MAC[14] *etc*. are the examples of scheduled/synchronous protocols. All these protocols maintain fixed wake-up interval and fixed listen period for communication. The major drawback of these protocols are the per-hop delay, which is equal to their wake-up interval. Although, T-MAC, R-MAC, and DW-MAC optimize the end-to-end delay by allowing the packet transmission to several number of hops within one wake-up interval, however, these protocols can not guarantee the latency requirement of delay intolerant applications. Moreover, due to the synchronous listen period among the neighbors, these protocols suffer very high contention and hence packet losses, and also not suitable for applications which require higher reliability. Providing the staggered schedule up to the sink, D-MAC reduces latency to a great extent, although it suffers high contention and packet losses, also lacks the provision of differentiated service.

The asynchronous protocols such as B-MAC [7], X-MAC [8] usually employ preamble-based transmission approach, in which long preamble is broadcasted for the duration equal to the wake-up interval of the receiver before actual packet transmission. These protocols suffer the per-hop delay which is on average equal to half of the wake-up interval. Although X-MAC introduces short preamble to reduce the per-hop latency, also the overhearing caused by long preamble, but concerning the QoS guarantee this protocols still suffer huge packet losses and large end-to-end delay due to the unnecessarily higher medium occupancy for preamble transmission. RI-MAC [9] and [15] are the recent asynchronous protocols that avoids the preamble based transmission through introducing receiver initiated transmission concept. Optimized for lower medium occupancy and hence, lower latency and reduced packet contention than preamble based approach, however, the per hop latency of RI-MAC is still on average half of the wake-up interval which in turn lengthen the end-to-end latency, also it can not ensure the higher reliability. In case of LET-MAC, although, it reduces the delay considerably as the traffic load increases, however, the end-to-end latency significantly increases during low traffic load. Moreover, it can not provide reliable services for loss-intolerant application.

Although some protocols both in synchronous and asynchronous approaches tries to reduce the latency and improve the reliability with some optimization, none of these protocols provide QoS guarantee in terms of both delay and reliability. Moreover, these protocols can not provide differentiated service to the applications based on their requirements. Offering some precise quality of service for diverse applications is first introduced in Q-MAC [10]. Q-MAC employs intra-node scheduling among different priority packets using MAX-Min fairness and packetized GPS algorithm to choose the next packet to be served from multiple queue within a node. It also performs inter-node scheduling employing the power conservation MACAW protocol and loosely prioritized random access protocol to allow transmission of packets among the nodes based on the their transmission urgency. Although it provides some service differentiation both within intra-node and inter-node, Q-MAC is not basically a duty-cycle MAC protocol and the energy efficiency is performed through reducing the collision probability and idle listening by providing differentiated contention period for different priority packets.

PQ-MAC [11] is the first protocol which integrates the QoS solution with synchronous duty cycle approach. It also defines several priority levels to provide differentiated service among diverse traffic. PQ-MAC introduces doubling scheme for the fast transmission of higher priority packets, in which nodes having more priority levels of data doubles its listen time within the sleep period and more transmission opportunity is given to the higher priority data than lower priority. Although the doubling scheme reduces the end-to-end latency of high priority traffic significantly compared with the other synchronous protocols, it can not ensure the meeting of certain delay deadline. Moreover, the doubling scheme works only if a node has more diverse priority data rather considering the priority of traffic itself. PQ-MAC also can not ensure the higher reliability for loss-intolerant traffic.

In [12], authors presented a QoS-MAC protocol for wireless multimedia sensor networks through dynamically changing the duty cycle and contention window according to the amount of dominating traffic classes after a certain time interval. This work also optimizes the delay for multimedia traffic and provides no guarantee of meeting certain delay deadline and reliability for the traffic with higher QoS requirements.

There also exists some TDMA based protocols [16,17] for WSNs. However, these protocols are mainly optimized for energy efficiency and providing collision free transmission, without any consideration of differentiated QoS among diverse applications, also optimizing the latency.

In Table 1, we summarize the addressed QoS metrics and differentiated service provided by the state-of-the-art duty cycle MAC protocols. We found no single MAC that addresses both the QoS metric along with differentiated service support. Hence the lack of any de-facto standard of duty-cycle based MAC providing differentiated multi-constrained QoS among diverse applications motivated us to devise MQ-MAC.

## 3. Preliminaries

### 3.1. Network System Models

We consider a set of *n* sensor nodes, ℕ = {*n*_1_, *n*_2_,..., *n_n_*} are deployed in a two-dimensional field and node *n*_0_ is a special node acting as a sink node of the network. We assume every node *n_i_* uses fixed transmission power for the communication with neighboring nodes. Thus, we model the network as, **G** = (ℕ, 𝔼), where 𝔼 is the set of all possible communication links. We assume the interference range of a node is twice of their transmission range. We further assume that, a subtree **T** from **G**, rooted at the sink has already been constructed by any existing routing algorithm, where some nodes in **T** act as source nodes (which generate data), some as forwarding nodes (forward other node’s data) or some play both the roles. Nodes forward data to the sink in a multi-hop manner forming many-to-one routing paradigm. We consider the nodes are static which implies the longevity of the routing tree.

We assume each node *n_i_* in T has a unique *id* and nodes know their corresponding *level* within **T**. The *kth* level of **T** is denoted as *l_k_* which determines the distance from root in hop count and a node can retrieve this information during network initialization (by broadcasting a control packet started from sink) or during route creation if shortest path routing protocol is used. We also denote the level of *n_i_* as *l_n_i__*. In tree topology, communication is only allowed between a parent node and its corresponding child nodes. We denote the parent of *n_i_* as *p_n_i__* and child of *n_i_* as *c_n_i__*. We define the *non-parent-child neighbor* of *n_i_*, denoted as *s_n_i__*, as the node, with which *n_i_* has communication link but is neither its parent nor child. In general, we define the neighbor set of *n_i_* denoted as *N*(*n_i_*) are the nodes with which *n_i_* has communication link. It includes *p_n_i__*, *c_n_i__* and *s_n_i__*. We consider all the communication links are symmetric, that is, if *n_i_* ∈ *N*(*n_j_*), then *n_j_* ∈ *N*(*n_i_*).

MQ-MAC requires time-synchronization among nodes. Implementation of the existing works [18,19] can meet the synchronization requirements in MQ-MAC. We can also adopt the global schedule algorithm (GSA) [20] which allows a large network to converge on a single global schedule.

### 3.2. Traffic Classification

MQ-MAC is designed for a WSN, which supports applications with diverse QoS requirements. In this paper, concerning both delay and reliability as QoS constraints, we classify the traffic as follows:

- *Class 0: Delay intolerant, loss intolerant traffic*. This type of traffic class demands the data delivery to the sink within certain delay bound as well as possesses higher reliability requirements (*i.e.*, near 100% reliability). For instance, distributed control applications fall into this category such as control systems in car manufacture or mobile robot control.
- *Class 1: Delay intolerant, loss tolerant traffic*. This traffic category requires timely delivery of data with certain bounds, however, it can tolerate some sort of packet losses. Example of this category includes monitoring applications whose monitored phenomenon is characterized by spatial correlation such as forest fire detection.
- *Class 2: Delay tolerant, loss intolerant traffic*. The traffic belongs to this class holds higher reliability requirements with no constraints on delay. This includes some critical-monitoring applications which requires some off-line post processing such as structural health monitoring application.
- *Class 3: Delay tolerant, loss tolerant traffic*. The traffic which has no constraints on both delay and reliability belong to this class. Examples include non-time critical monitoring applications such as agricultural application.

Notably, we consider only unicast traffic for traffic classification.

### 3.3. Queuing Model

**Figure 1.**
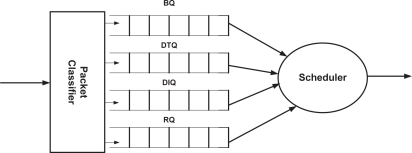
Queuing model.

Figure 1 illustrates the queuing model of MQ-MAC. Every node *n_i_* is equipped with a packet classifier which classifies the packet based on its type and QoS requirements. We provision four different types of queues to store diverse packets at MAC layer, namely, *broadcast queue* (BQ), *delay-tolerant queue* (DTQ), *delay-intolerant queue* (DIQ) and *retransmit queue* (RQ). The BQ is a priority queue which stores all types of broadcast packets but considering the packets used for synchronization as the highest priority and setting equal priority level for all other broadcast packets. DTQ is a FIFO queue which stores the delay tolerant (Class 2 and Class 3) packets and DIQ is a priority queue having delay intolerant (Class 0 and Class 1) packets. DIQ prioritizes the delay intolerant packets based on their remaining time to deadline. We adopt the approach in [21] for updating the value of remaining time to deadline. RQ stores the packets to be retransmitted of class 0 and class 2 only, since in MQ-MAC we make no provision for retransmitting loss-tolerant packets. RQ is a priority queue which prioritizes class 0 traffic over Class 2 traffic. Also, class 0 traffic with less remaining time to deadline is given higher priority in RQ. Every node also possesses a scheduler which schedules the packets to be transmitted at their respective transmission period.

Provisioning multiple queues does not increase the memory overhead considerably, since our queuing model just split different packet types in the separate queues instead of storing those in a single queue. Moreover, the recent sensor motes such as mica2 have sufficient on-board memory [22] which can easily afford the required memory requirements in our protocol. For instance, let the size of each queue is 10 packets and size of data packet is 50 bytes. (We used these values in our simulation as discussed in Section 5). Hence, the maximum memory requirement for MQ-MAC will be 2 KB, whereas, the on-board memory of mica2 mote is 4 KB.

## 4. MQ-MAC Protocol Details

MQ-MAC is a scheduled duty-cycle based MAC protocol which combines a hybrid approach in wake-up scheduling, including both synchronous and asynchronous. In *synchronous scheduled wake-up* approach, all nodes in a neighborhood wake up at the same time for communication while *asynchronous scheduled wake-up* approach allows nodes to wake-up asynchronously from its neighbors to receive packets but only the corresponding senders are scheduled to wake-up with the wake-up time of their receivers for sending packets. Both the approaches have distinct features which have impact on the QoS constraints. Hence, in MQ-MAC, nodes transmit packets in different wake-up approaches based on the multi-constrained QoS requirements of the packets.

### 4.1. Synchronous Scheduled Wake-up (SSW)

**Figure 2.**
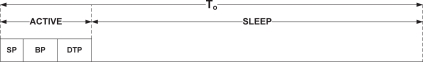
Synchronous scheduled wakeup.

In this approach, every node *n_i_* maintains a fixed operational cycle following the trend of existing scheduled approaches. An operational cycle, denoted as *T_o_* can be divided into two main periods: An ACTIVE period, denoted as *T_a_* and a SLEEP period, denoted as *T_s_*. The ACTIVE period is further divided into three periods: Synchronization period (SP), Broadcast period (BP) and Delay Tolerant Period (DTP). Figure 2 depicts the basic SSW approach. During SP, nodes broadcast SYNC packets to synchronize with the neighbors. The BP period is used to exchange other broadcast packets, for example, packets for route discovery, neighbor discovery, network wide queries, information dissemination *etc*. Nodes transmit delay tolerant packets (*i.e.*, both Class 2 and Class 3 packets) during DTP period. In MQ-MAC, the ACTIVE period and its constituent periods (SP, BP and DTP) are usually fixed according to the physical and MAC layer parameters. (*i.e.*, the channel bandwidth, packet size and contention window size). The SLEEP period may vary depending upon the length of *T_o_*.

**Figure 3.**
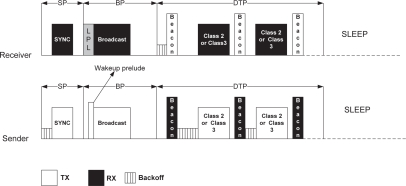
Medium access and packet transmission in SSW.

Nodes employ the basic CSMA/CA mechanism throughout the ACTIVE period to transmit SYNC packets, broadcast packets and the delay tolerant packets. Figure 3 exhibits the medium access mechanism, also the packet transmission in SSW. During SP, a node wakes up to transmit a SYNC packet if the period lies within the *synchronization interval* (time interval of broadcasting SYNC packets to maintain synchronization); otherwise it wakes up at the beginning of BP. In [23], authors presented an analytical model on deriving the optimal synchronization interval. Before transmitting SYNC packets, a node performs carrier sense to check channel activity after backing off by some duration. SYNC packets are transmitted immediately after finding the channel idle. Since, broadcast packets may not be transmitted at each operational cycle, hence, nodes perform low power listening (LPL) at the beginning of each BP. A node having broadcast packet in BQ sends a short *wake-up prelude* after finding the channel idle and then transmit the broadcast packet. Upon detecting channel activity, a receiving node powers up and receives the broadcast packet.

Although both SP and BP are used broadcasting, we differentiate the two periods for two reasons: *First*, Provisioning distinct periods for both SYNC and other broadcast packets improves the packet reliability. *Second*, Partitioning the two periods allows nodes to sleep for SP period if this is not the synchronization interval, and instead of fully listening the channel, a node performs LPL during BP because of the uncertainty of broadcast packet transmission at each operational cycle, thus saving energy.

MQ-MAC allocates DTP period for the transmission of delay tolerant packets in the SSW approach, since, in this approach, the per-hop latency is at least equal to the length of an operational cycle. Hence, in multi-hop scenarios, a packet might suffer large end-to-end delays, although the delay tolerant properties of this traffic class inhibits any performance degradation due to this large delay. In this period, MQ-MAC handles both loss tolerant and loss intolerant traffic along with their delay tolerant capabilities. But one of the shortcomings of synchronous wake up approach is that, nodes might suffer huge packet collision, since all the nodes in a neighborhood having data contend for the channel at the same time. However, provisioning distinct period only for delay tolerant packets reduces the channel contention probability to a great extent, although this does not guarantee 100% packet delivery for Class 2 traffic. Hence, the lost packets of Class 2 traffic will be retransmitted during the asynchronous wake up approach which provides collision free transmission as to be discussed later.

We adopt the receiver-initiated [9] approach for unicast packet transmission. In receiver-initiated approach, data transmission begins at the sender after the beacon packet reception, which is transmitted by the receiver. Hence, during DTP, a node, while functioning as a receiver (*i.e.*, having no packets in DTQ), transmits a beacon packet after performing backoff followed by a clear channel assessment (CCA) check. The sender (*i.e.*, nodes having data in DTQ) on the other hand, start random backoff within a fixed contention window after beacon reception. The wining sender then start transmitting packet if the channel is found idle. The losing senders freeze their backoff to get higher priority of channel access for the next contention in the same DTP. The receiver acknowledges the reception of data packet by another beacon packet which also serves as an invitation to the potential senders to send their pending packets. The process continues until the DTP ends. The senders goes to sleep immediately either if its DTQ remains empty or DTP ends. In contrast, receivers turns into sleeping state if it does not receive any data packets until waiting for maximum backoff period after the sending of beacon packet. A receiver also goes to sleep after DTP ends.

During DTP, we make no provision for packet retransmission, since, retransmission of loss intolerant packets will be handled by the asynchronous scheduled wake up approach and the loss tolerant features of Class 3 packets is sufficient for maintaining certain delivery ratio without retransmission, since assigning a separate period for delay tolerant packets only greatly reduces the contention probability.

### 4.2. Asynchronous Scheduled Wake-up (ASW)

In asynchronous scheduled wake-up approach of MQ-MAC, the node wakes up at different times than its neighbors for packet reception. This approach utilizes the SLEEP period of synchronous scheduled wake-up for packet transmission. In particular, after the ACTIVE period ends, nodes again wake up asynchronously at a particular time during the SLEEP period of *T_o_*, to receive data packets from the intended senders. This approach is mainly used to transmit packets which have both delay and loss intolerant properties (*i.e.*, Class 0, Class 1 and retransmitted packets of Class 2). Hence, we introduce a distributed *reception-based TDMA scheduling (RTDMA)* mechanism which assigns collision free and delay-efficient reception slot to a node *n_i_* ∈ **T**.

**Figure 4.**
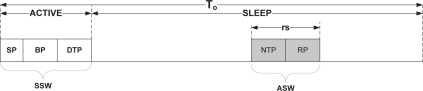
Asynchronous scheduled wake-up.

Figure 4 shows the basic ASW approach. In this approach, we assume that the SLEEP period of *T_o_* is logically divided into *reception slots*, *rs* ∈ {0, 1, 2....*K* – 1}. In each reception slot, a node receives multiple data packets from its corresponding child nodes. The size of the reception slot denoted as, *T_slot_* determines the maximum number of packets a node can receive from its children including retransmitted packets. The reception slot is thereby contain two periods: New Transmission Period (NTP) and Retransmission Period (RP). During NTP, nodes receive only newly transmitted Class 0 and Class 1 packets from its children while at RP, nodes retransmit the lost packets of Class 0 and Class 2 to their parents. We allocate distinct period for retransmitted packets to avoid the channel contention with the newly transmitted packets.

*T_slot_* is a configuration parameter which is determined by the sink node during network initialization phase. Sink determines the NTP length based on the maximum number of delay-intolerant flow in the network, the corresponding beacon packet number, and the physical and MAC layer parameters. (*i.e.*, channel bandwidth, contention window size, data and beacon packet length). On the other hand, sink may also choose the RP length through the information of the packet loss rate for Class 0 and Class 2 packets of the bottleneck node, the corresponding beacon packet number, and the physical and MAC layer parameters.

#### 4.2.1. Meeting the Delay Constraint

MQ-MAC aims to provide the desired delay guarantees for delay intolerant packets. MQ-MAC satisfies the goal in two ways: *first,* choosing the appropriate *T_o_* length, and *second*, assigning the reception slots during the SLEEP period of *T_o_* in such a way that all the delay intolerant packets can be reached from sources to the sink within the SLEEP period of *T_o_*. We discuss about the choosing of *T_o_* in Section 4.3 as presented later.

To assign delay-efficient reception slots, we assume that, a routing tree **T** already exists in the network. This avoids the random reception scheduling of nodes which could introduce high latency. To illustrate this, consider the schedule order is chosen as the opposite of the routing path order (*i.e.*, sink is scheduled first and source is last). This leads to the packet latency *d* operational cycle where *d* is the path length from source to sink. Hence, we assign the reception slots that satisfy the following constraint:

(1) $${\mathrm{rs}}_{p_{n_{i}}}>{\mathrm{rs}}_{n_{i}}$$

Equation 1 implies that, reception slot of the parent of *n_i_* must be greater than the reception slot of *n_i_* itself. Assigning the reception slot in such a way limits the maximum end-to-end delay bound of the delay intolerant packets equal to the SLEEP period from the starting time of packet transmission for all the sources with an assumption that SLEEP period is long enough to accommodate the reception slots of all nodes in **T**.

#### 4.2.2. Meeting the Reliability Constraint

The loss-intolerant applications require very high reliability (near to 100% delivery ratio) which necessitates a collision free data transmission mechanism. In spite of having lower design complexity and resilient to network changes, the existing CSMA/CA mechanism does not guarantee a collision free transfer in a multi-hop scenario due to the hidden node problem even with the use of RTS/CTS [24]. Hence, TDMA is the only choice for ensuring higher reliability of the loss-intolerant applications although it incurs some protocol overhead.

The existing distributed TDMA-based MAC protocols for sensor network [16,17] maintains two-hop neighborhood information to choose collision free transmission slot resulting high memory overhead. The situation could be even more worse in case of dense sensor network. Considering the pitfalls of the state-of-the-art TDMA protocols, our proposed RTDMA possesses the following properties:

- Given a routing tree, **T**, RTDMA schedules the node’s reception slot at each level *l_k_* instead of transmission for a simplified scheduling, avoiding the maintenance of transmission schedule information of all two-hop neighborhood to a great extent.
- Node’s reception schedules are chosen in such a way that, it provides interference-free reception for ensuring higher reliability.
- We provision for multi-packet reception at each reception slot from the corresponding child nodes to avoid the multiple wake-up in an operational cycle thus saving energy, also avoiding the reception slot assignment for each of the child node.

Although the reception slots are scheduled based on TDMA, we allow CSMA like approach for transmitting data by the children nodes to their parent node. Devising an interference free reception schedule eliminates the collision probability with the interfering nodes. Thus the basic CSMA procedure suffices for ensuring collision free transmission.

#### 4.2.3. Reception-Based TDMA Scheduling (RTDMA)

The proposed RTDMA mechanism contains two components: *interfering-receiver table construction* and *slot-assignment*. The details of these two components are as follows:

***Interfering-receiver table construction*** This component constructs a table containing the interfering-receiver of a node of the same level as defined below.

**Definition 1.** *Consider two nodes n_i_ and n_j_ are in l_k_ of a routing tree* **T***. Assume that, only nodes in l*_*k*−1_ *(the next lower level nodes) are transmitting to the nodes in l_k_. We define the n_i_ and n_j_ are interfering-receiver to each other if,*

1. transmission of any child of node n_i_ interferes at n_j_ while n_j_ is receiving from its children or vice-versa. and
2. transmission of any non-parent-child neighbor of n_i_ to n_j_ interferes at n_i_ while it is receiving from its children.

Both the condition of being interfering-receiver given in the definition satisfies if the following relationship exists between two nodes *n_i_* and *n_j_* (both are in same level, *l_k_*) and any of their corresponding child node *c_n_i__* and *c_n_j__*.

1. *n_i_* and *n_j_* are non-parent-child neighbor to each other.
2. *c_n_i__* and *c_n_j__* are non-parent-child neighbor to each other.
3. *c_n_j__* is the non-parent-child neighbor of *n_i_*.

**Figure 5.**
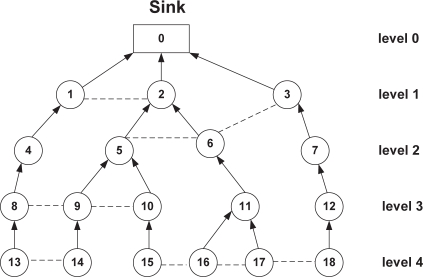
Routing tree for 19 nodes where node 0 is the sink. The arrow indicates the parent-child relationship and the dotted line indicates the communication link with non-parent-child neighbor.

To illustrate this, consider the routing tree as shown in Figure 5. Here, node 1, 2 and 3 all are in level 1 of the tree. In this case, node 1 and 2 are interfering-receiver since, transmission of any child of node 2 (*i.e.*, node 5 and node 6) interferes with node 1 (since, both node 5 and 6 are the two-hop neighbors of node 1 and hence in the interference range), while node 1 is receiving data from its child node. (*i.e.*, node 4) and vice-versa. Notably, node 1 and node 2 both are non-parent-child neighbor to each other (*i.e.*, first relationship). Similarly, node 10 and node 11 of level 3 are also interfering receiver and in this case node 15 (child of node 10) and node 16 (child of node 11) are neighbor to each other (*i.e.*, second relationship). By definition, node 3 and node 2 are also interfering-receiver, since transmission of node 6 to its parent (node 2) interferes at node 3 while node 3 is receiving from its child node 7, where node 6 is the non-parent-child neighbor of node 3 (*i.e.*, third relationship). However, node 1 and 3 are non-interfering receiver at level 1, since none of the condition in the definition holds for these nodes.

To construct interfering-receiver table, every node runs a simplified neighbor discovery protocol. Unlike the existing TDMA protocols [16,17] a node does not broadcast all of its one-hop neighbor list. Considering the routing tree, **T**, and assuming the broadcasting node is in *l_k_*, a node embeds the following information in the broadcast packet: (i) Source address, (ii) ID of those neighbors which are in *kth* and (*k*−1)*th* level (upper level of *l_k_*). (iii) ID of the non-parent-child neighbor’s parent (a node obtains this information after receiving its non-parent-child neighbor’s neighbor information). In each of the cases, a node also includes the corresponding *l_k_* value with each ID.

**Algorithm 1:** Interfering-receiver table construction of node *n_i_*

1.	Given *T T_n_i__*
2.	**for all***n_j_* ∈ *N*(*n_i_*) such that 1 ≤ *n_j_* ≤ *M***do**
3.	**for all***n_k_* ∈ *I*(*n_j_*) such that 1 ≤ *n_k_* ≤ *N***do**
4.	**if***l_n_j__* ≠ *l_n_i__* and *l_n_k__* ≠ *l_n_i__***then**
5.	delete *n_j_* and *n_k_* from *TT_n_i__*
6.	**end if**
7.	**if***l_n_j__* = *l_n_k__* = *l_n_i__***then**
8.	delete *n_k_* from *TT_n_i__*
9.	**end if**
10.	**end for**
11.	**end for**

Every node *n_i_* then builds a temporary table *TT_n_i__* consisting two fields: The first field contains the one-hop neighbor information (ID and the corresponding *l_k_* value of *N*(*n_i_*)) and the second field contains the ID of the nodes it received from its one-hop neighbor along with their level value. We denote the set of nodes in the second field for a particular node *n_j_* ∈ *N*(*n_i_*) of the first field, as *I*(*n_j_*). After that, a node deduces the interfering-receiver table from *TT_n_i__*.

Algorithm 1 exhibits the construction of interfering-receiver table of a node *n_i_*. The algorithm takes *TT_n_i__* as an input to build the table. It searches every row of *TT_n_i__* to eliminate the irrelevant nodes (line 2 and 3). Here, *M* represents the number of rows in *TT_n_i__* (in other words, the number of neighbors of *n_i_*) and *N* represents the number of nodes in *I*(*n_j_*). Since, our main focus is to find out the interfering receivers which are in the same level of node *n_i_*, hence all the nodes with different level other than node *n_i_* are deleted from *TT_n_i__* (line 4 and 5). Furthermore, since, non-parent-child neighbors of *n_j_* which are in the same level of *n_i_* are non-interfering receiver to *n_i_*, thus those nodes are also deducted from *TT_n_i__*. Finally, the interfering-receiver table of *n_i_* contains only those nodes having same level of node *n_i_*, which are interfering to each other while receiving data from their corresponding child nodes in a same slot. We denote the set of nodes in the interfering receiver table of *n_i_* as *IR*(*n_i_*).

**Slot-assignment** The slot-assignment component assigns the reception slots of the nodes in **T** in a top-down approach. This component uses three packets for slot-assignment. The *Request* packet contains the desired slot number of a node *n_i_*. It also includes its number of interfering-receivers, denoted as $N_{n_{i}}^{\mathrm{IR}}$, where $N_{n_{i}}^{\mathrm{IR}}=\left|\mathrm{IR}\left(n_{i}\right)\right|$. The *Confirm* packet incorporates the confirmed slot number of *n_i_* and the *Cancel* packet of a *n_i_* includes the slot number to be canceled by a node *n_j_*. Node *n_i_* broadcasts its *Request*, *Confirm* and *Cancel* packets to its neighbors. Besides, it broadcasts the corresponding packets of those one hop neighbors which are in the same level or upper level of *n_i_* (*i.e.*, *kth* and (*k* − 1)*th* level, assuming *n_i_* is in *l_k_*), also the packets of its non-parent-child neighbor’s parent. We describe the slot-assignment procedure as follows:

1. Initially, sink sets its reception slot as *K* −1 (*i.e.*, the last slot of *T_s_*) and broadcasts it to all of its neighbors.
2. A node *n_i_* ∈ *l_k_* having no slot-assigned so far temporarily sets its reception slot, *rs_n_i__* as
  (2) $${\mathrm{rs}}_{n_{i}}={\mathrm{MSN}}_{k-1}-1$$
  Here, *MSN*_*k*−1_ denotes the maximum slot number already occupied by the nodes in (*k* − 1)*th* level. A node obtains the value of *MSN*_*k*−1_ through the *Confirm* packets. Hence, before setting the tentative value of *rs_n_i__*, it is necessary to receive the occupied slot information of the upper level nodes. Every node except the nodes in level 1 thus waits for a period of time *T_w_* before setting the *rs_n_i__*, tentatively. The value of *T_w_* should be proportional to *D*^2^, where *D* is the maximum number of neighbors of a node, *n_i_* ∈ **T** in its same level and upper level.
3. Node *n_i_* stores the confirmed slot number of the nodes which are in its interfering-receiver table(through the information obtained from the *Confirm* packet). *n_i_* then updates its *rs_n_i__* as
  $${\mathrm{rs}}_{n_{i}}=\{\begin{array}{ll} {\mathrm{rs}}_{n_{i}}-1, & \mathrm{if}\;({\mathrm{rs}}_{n_{i}}={\mathrm{rs}}_{n_{j}});\exists_{n_{j}},\;\mathrm{where},n_{j}\in \mathrm{IR}(n_{i})\\ \mathrm{UNCHANGED}, & \mathrm{otherwise}. \end{array}$$
4. *n_i_* embeds the *rs_n_i__* in its *Request* packet and broadcasts it to its neighbors. It then waits for *T_w_* period.
5. During *T_w_*, if *n_i_* receives a *Cancel* packet, it decrements *rs_n_i__* by 1 and starts from step 3 again. After waiting for *T_w_*, if no *Cancel* packet arrives including ID of node *n_i_* in it, the node then broadcasts the *Confirm* packet.
6. If *n_i_* receives a *Confirm* packet from node *n_j_* where *n_j_* ∈ *IR*(*n_i_*), then it goes back to step 3. If it receives a *Request* packet from *n_j_*, where *l_n_i__* = *l_n_j__* and *rs_n_i__* = *rs_n_j__* and *n_j_* ∈ *IR*(*n_i_*), it broadcast a *Cancel* packet if (a) *n_i_* already broadcasted its *Confirm* packet, or (b) $N_{n_{i}}^{\mathrm{IR}}>N_{n_{j}}^{\mathrm{IR}}$ or (c) (*n_i_* > *n_j_*) *and* $\left(N_{n_{i}}^{\mathrm{IR}}=N_{n_{j}}^{\mathrm{IR}}\right)$

**Figure 6.**
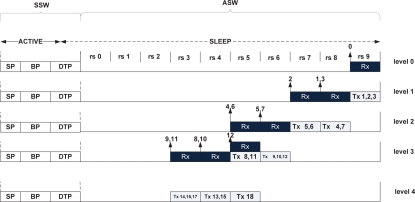
Slot assignment of RTDMA.

Figure 6 exhibits the slot assignment of RTDMA based on the routing tree as shown in Figure 5. In this figure, the black rectangle represents the reception slot of a node in a particular level of the tree and the white rectangle stands for the transmission period of the nodes in that level. As shown in this figure, RTDMA assigns only the non-interfering receiver nodes the same reception slot either in the same level (*i.e.*, node 4 and node 6 of level 2) or in different level (*i.e.*, node 4 or node 6 of level 2 and node 12 of level 3).

***Correctness of RTDMA*** RTDMA exhibits correctness if the packet reception of the nodes is collision free due to interference. It necessitates the assignment of reception slot of the nodes are done as interference-free.

In RTDMA, the reception slot, *rs_n_i__* for node *n_i_* is assigned following two constraints:

1. Let *n_j_* be the node which is in same level of *n_i_* and *n_j_* ∈ *IR*(*n_i_*). Hence, *rs_n_i__* ≠ *rs_n_j__*.
2. The *rs_n_i__* must be at least one slot ahead of the maximum occupied slot number of the upper level nodes which is observed by *n_i_*.

The first constraint ensures that, the slot assignment procedure will never select the same slot for two interfering receiver at the same level and hence, the simultaneous reception of two nodes at the same level must be interference free, assuming the interference might occur only for the transmission by the next lower level nodes. (refer to the definition of interfering-receiver).

The second constraint satisfies the following theorem.

**Theorem 1.** *Given a routing tree,* **T***. The slot assignment procedure assign slots for nodes in* **T** *such a way that, two nodes in different level will have same reception slot only if their reception are interference-free.*

*Proof.* We prove the theorem in two steps. *First,* we prove that, two nodes in the consecutive level will have same reception slot if their reception are interference free and *second* we prove that if same reception slot is selected using the slot assignment procedure by any two nodes which are not in the consecutive level, their reception are also interference free. Arguing for the first step, assume two node *n_i_* and *n_j_*, where *n_i_* is in *l_k_* and *n_j_* is in *l*_*k*−1_, that is *n_j_* is in the upper level of *n_i_*. Also, assume that the slot-assignment procedure allocates the reception slot of two nodes such that, *rs_n_i__* = *rs_n_j__*. During slot assignment, *n_i_* observes the maximum slot number already occupied by the nodes in *l*_*k*−1_ through the reception of *Confirm* packets broadcasted by the nodes in *l*_*k*−1_. Here, *n_i_* receive the *Confirm* packets from all of the nodes in *l*_*k*−1_ which are at least two-hops away. It implies that *n_i_* will receive the slot information of all of its neighbor’s (which are at *l_k_*) parent. Besides, it receives packets from a node *n_k_* ∈ *l*_*k*−1_ which is three-hop away from *n_i_* but one of the child of node *n_k_* is within two-hop neighborhood of node *n_i_*. Therefore, if *rs_n_i__* = *rs_n_j__*, it signifies that, there will be no children of node *n_j_* acting as a transmitter which are within the two-hop neighborhood of node *n_i_*. Hence, the reception of both node *n_i_* and *n_j_* will be interference-free.

We prove the second case by contradiction. For this, assume three nodes *n_i_*, *n_j_* and *n_k_* where, *n_i_* is in *l_k_*, *n_j_* is in *l*_*k*−1_ and *n_k_* is in *l*_*k*−2_ and *rs_n_i__* = *rs_n_k__*. Assuming the avoidance of interference by all other factors at *n_i_*, in this case, *n_i_*’s reception will be interfered only if *n_j_* is the child of node *n_k_* and *n_j_* is within two-hop neighborhood of *n_i_*. Hence, if *n_j_* is the child of node *n_k_*, then reception slot of *n_j_* will be at least one slot ahead of node *n_k_* (second constraint). Moreover, if *n_j_* is within the two-hop neighborhood of *n_i_*, then, *n_i_*’s reception slot also must be at least one slot ahead of *n_j_* (second constraint). Therefore, our slot assignment procedure will never assign the same reception slot for *n_i_* and *n_k_*. On the other hand, assuming the avoidance of interference by all other factors at node *n_k_*, if *rs_n_i__* = *rs_n_k__*, then node *n_k_*’s reception will not be interfered by the transmitter of *n_i_* since, the transmitter of *n_i_* must be at least three hop away from node *n_k_*. Hence, if two nodes are not in the consecutive level but having same reception slot, then their reception will also be interference-free and this completes the proof.

#### 4.2.4. Access Method

In ASW, a node *n_i_* wakes up at the beginning of its assigned reception slot (also the start time of NTP) and immediately transmits a beacon packet after finding the channel idle, to receive delay intolerant packets from its children. The corresponding child nodes having packets in DIQ wake up a bit earlier than its parent’s NTP start time and perform LPL. The access method and packet transmission during NTP of ASW follows almost similar procedure as DTP of SSW. However, there are some differences between these two: *first*, the receivers perform backoff before transmitting beacon during DTP while receivers in NTP of ASW immediately transmits beacon after wake up, since in this period, only one receiver transmits beacon within its two-hop neighborhood. *Second*, nodes in NTP of ASW choose the contention window value for the head of line (HOL) packet which is proportional to the value of remaining time to the deadline or a maximum contention window value (*i.e.*, lower the remaining time to the deadline, lower the contention window value). *Third*, The nodes in DTP freezes their backoff if they lose contention for a particular DTP, however, nodes in NTP always starts from new backoff after getting beacon for a particular NTP of ASW.

During RP, a node *n_i_* transmits beacon containing a bit which indicates an invitation of sending retransmitted packet from RQ. If any child node of *n_i_* has no packets in DIQ during NTP but having packets in RQ, then instead of waking up at the start of NTP, it wakes up a bit earlier than its parent’s RQ and waits for parent’s beacon while performing LPL. The access method and packet transmission procedure are also similar to NTP except the contention period value chosen for Class 2 packets in which it is set as the default contention value of DTP.

A receiver in NTP and RP goes to sleep mode if no data packet comes after waiting for maximum backoff period and wakes up again fully after NTP ends. A sender in NTP goes to sleep if it has no remaining packets both in DIQ and RQ. However, if it has packets in RQ but its DIQ became empty during NTP, it turns into LPL and waits until it gets parent’s (*i.e.*, receiver) beacon while RP starts. A sender also goes to sleep after RP ends.

### 4.3. Analysis

This section presents an analysis of the energy and latency for MQ-MAC protocol.

A. *Energy analysis*. In this analysis, we focus on the radio energy consumption in wireless sensor nodes, as it is the most dominant source of energy consumption for WSNs [2]. Typically, a radio has four different states: listen, transmit, receive, and sleep; the power consumption of each can be denoted as *P_l_*, *P_t_*, *P_r_*, and *P_s_*. Since channel polling is different from typical listening [23], we present the energy consumption for polling, *P_p_*, separately. We can measure the energy consumption of a radio device by determining the time it stays in each state, denoted as *T_l_*, *T_t_*, *T_r_*, *T_p_*, and *T_s_*. Thus, the expected energy consumption per node for MQ-MAC can be modeled as

(3) $$\begin{array}{ll} E_{\mathrm{MQ}-\mathrm{MAC}} & =E_{t}+E_{r}+E_{l}+E_{p}+E_{s}\\ & =P_{t}T_{t}+P_{r}T_{r}+P_{l}T_{l}+P_{p}T_{p}+P_{s}T_{s} \end{array}$$

In this analysis, we use the power values of Mica2 CC1000 radio [23] for actual representation of the model, as shown in Table 2. Since, the *P_s_* is much smaller than the other power values, we omit this from Equation 3 and modeled the energy consumption considering the other power values. Thus, the Equation 3 becomes

(4) $$E_{\mathrm{MQ}-\mathrm{MAC}}=P_{t}T_{t}+P_{r}T_{r}+P_{l}T_{l}+P_{p}T_{p}$$

(5) $$\begin{array}{ll} T_{t}= & \underset{T_{t}^{\mathrm{SP}}}{\underset{︸}{R_{\mathrm{syn}}L_{\mathrm{syn}}}}+\underset{T_{t}^{\mathrm{BP}}}{\underset{︸}{R_{\mathrm{br}}\left(L_{\mathrm{br}}+L_{\mathrm{pl}}\right)}}+\underset{T_{t}^{\mathrm{DTP}}}{\underset{︸}{\left(R_{\mathrm{dtp}}^{o}+R_{\mathrm{dtp}}^{f}\right)L_{\mathrm{dtp}}+\frac{L_{b}}{T_{o}}+L_{b}R_{\mathrm{dtp}}^{f}}}+\underset{T_{t}^{\mathrm{NTP}}}{\underset{︸}{\left(R_{\mathrm{dip}}^{o}+R_{\mathrm{dip}}^{f}\right)L_{\mathrm{dip}}+\frac{L_{b}}{T_{o}}+L_{b}R_{\mathrm{dip}}^{f}}}\\ & \underset{T_{t}^{\mathrm{RP}}}{\underset{︸}{+R_{\mathrm{rp}}^{t}L_{\mathrm{dtp}}+\frac{L_{b}}{T_{o}}+R_{\mathrm{rp}}^{r}L_{b}}} \end{array}$$

(6) $$\begin{array}{ll} T_{r}= & \underset{T_{r}^{\mathrm{SP}}}{\underset{︸}{N\left(R_{\mathrm{syn}}L_{\mathrm{syn}}\right)}}+\underset{T_{r}^{\mathrm{BP}}}{\underset{︸}{N\left(R_{\mathrm{br}}\left(L_{\mathrm{br}}+L_{\mathrm{pl}}\right)\right)}}+\underset{T_{r}^{\mathrm{DTP}}}{\underset{︸}{R_{\mathrm{dtp}}^{f}L_{\mathrm{dtp}}+\frac{L_{b}}{T_{o}}+\left(pR_{\mathrm{dtp}}\right)L_{b}}}+\underset{T_{r}^{\mathrm{NTP}}}{\underset{︸}{R_{\mathrm{dip}}^{f}L_{\mathrm{dip}}+\frac{L_{b}}{T_{o}}+R_{\mathrm{dip}}L_{b}}}\\ & +\underset{T_{r}^{\mathrm{RP}}}{\underset{︸}{R_{\mathrm{rp}}^{r}L_{\mathrm{dtp}}+\frac{L_{b}}{T_{o}}+R_{\mathrm{rp}}^{t}L_{b}}} \end{array}$$

(7) $$T_{l}=\underset{T_{l}^{\mathrm{SP}}}{\underset{︸}{\frac{\mathrm{SP}}{T_{o}}(1-T_{t}^{\mathrm{SP}}-T_{r}^{\mathrm{SP}})}}+\underset{T_{l}^{\mathrm{BP}}}{\underset{︸}{\frac{\mathrm{BP}}{T_{o}}(1-T_{t}^{\mathrm{BP}}-T_{r}^{\mathrm{BP}})-T_{p}}}+\underset{T_{l}^{\mathrm{DTP}}}{\underset{︸}{T_{\mathrm{bo}}(1+R_{\mathrm{dtp}})}}+\underset{T_{l}^{\mathrm{NTP}}}{\underset{︸}{T_{\mathrm{bo}}R_{\mathrm{dip}}}}+\underset{T_{l}^{\mathrm{RP}}}{\underset{︸}{T_{\mathrm{bo}}(R_{\mathrm{rp}}^{r}+R_{\mathrm{rp}}^{t})}}$$

(8) $$T_{p}=\underset{T_{p}^{\mathrm{BP}}}{\underset{︸}{\frac{\mathrm{BP}}{T_{o}}T_{w}^{\mathrm{br}}}}$$

Equations 5–8 delineates the expected staying time of a node in different states. Table 3 shows the different parameter of MQ-MAC along with their meaning used in this analysis. Here, we assume that, all the packets of a particular type have fixed length. All the packet sizes in this table are expressed in transmission time units. In all the equations from 5–8, the expected staying time is the combination of the times a node stays in different periods (*i.e.*, SP, BP, DTP, NTP and RP) of the corresponding state and the notation in the under-brace represents the expected staying time in the relevant periods.

In Equation 5 both $T_{t}^{\mathrm{SP}}$ and $T_{t}^{\mathrm{BP}}$ include the SYNC packet transmission time and broadcast packet (along with wake-up prelude) transmission time respectively. The $T_{t}^{\mathrm{DTP}}$ and $T_{t}^{\mathrm{NTP}}$ incorporate the total delay tolerant (DT) packet and delay intolerant (DI) packet transmission time respectively, including both originating and transit (packets received from the child nodes) packets. These also include the beacon packet transmission time at every *T_o_*, along the total ACK beacon transmission time for the successful reception of both DT and DI packets. Likewise, the $T_{t}^{\mathrm{RP}}$ includes the expected transmission time for the retransmitted packets and the beacon packet transmission time. Here, we assume the retransmission of only class 2 packets and no packet loss will occur during asynchronous wake up approach. Hence, for simplicity, we exclude the retransmission of class 0 packets as it is transmitted during ASW, also we assume only one retransmission suffices for the successful reception during RP of ASW. Thus, we measure the $R_{\mathrm{rp}}^{t}$ in $T_{t}^{\mathrm{RP}}$ as-

(9) $$R_{\mathrm{rp}}^{t}=\alpha (1-p)R_{\mathrm{dtp}}$$

where α is the ratio of class 2 packets and *p* is the probability of success of delay tolerant packets (both class 2 and class 3) and hence (1 – *p*) denotes the packet loss probability.

In Equation 6, the $T_{r}^{\mathrm{SP}}$ and $T_{r}^{\mathrm{BP}}$ include the SYNC packet and broadcast packet reception time from *N* neighbors. All of the $T_{r}^{\mathrm{DTP}}$, $T_{r}^{\mathrm{NTP}}$ and $T_{r}^{\mathrm{RP}}$ incorporates the expected reception time of DT packets ( $R_{\mathrm{dtp}}^{f}L_{\mathrm{dtp}}$), DI packets ( $R_{\mathrm{dip}}^{f}L_{\mathrm{dip}}$) and retransmitted packets ( $R_{\mathrm{rp}}^{r}L_{\mathrm{dtp}}$) from the child nodes respectively. Furthermore, these include the beacon reception time at each *T_o_*, also the total ACK beacon reception time by the sender for successfully transmission of DT packets, DI packets and retransmitted packets.

The $T_{l}^{\mathrm{SP}}$ and $T_{l}^{\mathrm{BP}}$ of Equation 7 represent the expected listening time during SP and BP, which is measured by deducting the expected transmission and reception time from the total SP and BP at each *T_o_*. Since, only the nodes in BP perform LPL, hence this fraction of time has also been subtracted to measure $T_{l}^{\mathrm{BP}}$. The $T_{l}^{\mathrm{DTP}}$ includes the expected listening time while performing beacon backoff, along with data backoff period for each data packet transmission time. Since, nodes in NTP does not perform beacon backoff, hence this has been deducted while measuring the $T_{l}^{\mathrm{NTP}}$. Following the same procedure, the $T_{l}^{\mathrm{RP}}$ includes the average backoff time a node perform listening the channel before transmitting data, also the idle listening performed by the receivers while the corresponding child nodes are in backoff before transmitting their packets.

Finally, *T_p_* in Equation 8 represents the expected polling time of a node only during BP at each *T_o_*, which includes the average waiting time for a broadcast packet reception.

B. *Delay analysis*. Since, MQ-MAC handles packets of diverse type based on their QoS requirements, here we present the end-to-end delay analysis for different types of packets. In this analysis, we assume the traffic load is very light and hence, no queueing delay will occur.

*Latency for SYNC, broadcast and delay tolerant packet*. The average end-to-end delay for SYNC packet, broadcast packet and delay tolerant packets can be formulated as

(10) $$E_{\mathrm{syn}}=\frac{T_{o}}{2}+\{\sum_{i=2}^{N}T_{o}\}+T_{\mathrm{bo}}+L_{\mathrm{syn}}$$

(11) $$E_{\mathrm{br}}=\frac{T_{o}}{2}+\{\sum_{i=2}^{N}T_{o}\}++\mathrm{SP}+T_{\mathrm{cs}}+L_{\mathrm{syn}}+L_{\mathrm{pl}}$$

(12) $$E_{\mathrm{dtp}}=\frac{T_{o}}{2}+\{\sum_{i=2}^{N}T_{o}\}+\mathrm{SP}+\mathrm{BP}+2T_{\mathrm{bo}}+L_{b}+L_{\mathrm{dtp}}$$

In Equations 10 to 12, $\frac{T_{o}}{2}$ represents the average delay incurred at the first hop. Since, at source node, packet may arrive at any time within a frame. For all other hops, the per-hop delay is equal to the length of *T_o_*. *T_cs_* in Equation 11 denotes the carrier sense delay. The other symbols used in Equations 10 to 12 carry the same meaning as given in Table 3.

*Latency for delay intolerant packet*. For delay intolerant packets, a packet can be propagated from source to the sink within the sleep time, *T_s_*, if the packet arrives at the source before its parent’s reception slot. The best case of end-to-end delay happens when the packet arrives just before the parent’s reception slot starts. In that case, the end-to-end delay will be

(13) $$E_{\mathrm{dip}}^{\mathrm{best}}=\mathrm{rs}\left(K-1\right)T_{\mathrm{slot}}-{\mathrm{rs}}_{p_{n_{s}}}T_{\mathrm{slot}}+T_{\mathrm{bo}}+L_{b}+L_{\mathrm{dip}}$$

where, *rs*(*K*−1) denotes the maximum reception slot number, *rs_p_n_s___* denotes the reception slot number of the parent of source node and *T_slot_* denotes the reception slot length.

The worst case end-to-end delay occurs when the packet arrives at a source node just after its parent reception slot ends. In that case, it requires about one operational cycle until the next reception slot of the parent of source node comes. Hence, the worst case delay will be

(14) $$E_{\mathrm{dip}}^{\mathrm{worst}}=T_{o}+\mathrm{rs}\left(K-1\right)T_{\mathrm{slot}}-{\mathrm{rs}}_{p_{n_{s}}}T_{\mathrm{slot}}-T_{\mathrm{slot}}+T_{\mathrm{bo}}+L_{b}+L_{\mathrm{dip}}$$

Here, one reception slot time is deducted since, *T_o_* includes the reception slot of the parent node of source.

Considering the packet may arrive at any time at the source node, the average end-to-end delay for the delay intolerant packet becomes

(15) $$E_{\mathrm{dip}}^{\mathrm{average}}=\frac{T_{o}}{2}+\mathrm{rs}\left(K-1\right)T_{\mathrm{slot}}-{\mathrm{rs}}_{p_{n_{s}}}T_{\mathrm{slot}}-T_{\mathrm{slot}}+T_{\mathrm{bo}}+L_{b}+L_{\mathrm{dip}}$$

C. *Choosing the T_o_*. We can choose the length of *T_o_* based on the worst case end-to-end delay analysis as given in Equation 14. Here, we choose the $E_{\mathrm{dip}}^{\mathrm{worst}}$ for the delay intolerant application which has the minimum end-to-end delay requirement. In Equation 14,*rs*(*K* − 1)*T_slot_* – *rs_p_n_s___* *T_slot_* − *T_slot_* + *T_bo_* + *L_b_* +*L_dip_* ≤ *T_s_*, since the reception slots are allocated within the *T_s_*. Thus, we get,

(16) $$\begin{array}{ll} E_{\mathrm{dip}}^{\mathrm{worst}} & =T_{o}+T_{s}\\ & =T_{o}+(T_{o}-T_{a})\\ & =2T_{o}-T_{a} \end{array}$$

Since, the value of *T_a_* is fixed, hence, from Equation 16 we derive the *T_o_* as

(17) $$T_{o}=\frac{E_{\mathrm{dip}}^{\mathrm{worst}}+T_{a}}{2}$$

However, in the absence of any delay-intolerant application, nodes might choose a fixed *T_s_* to obtain the value of *T_o_*, similar to the existing synchronous approaches.

## 5. Performance Evaluations

This section presents the performance evaluation of the MQ-MAC protocol. The results illustrate that MQ-MAC successfully achieves its design goals.

### 5.1. Simulation Environment

We perform extensive simulation of our protocol using the ns-2 network simulator. We used version 2.33 of the ns-2 simulator using the Two Ray Ground propagation model in the air and a single Omni-directional antenna commonly used with ns-2. A network of area 1,000 m × 1,000 m is used with 50 nodes deployment in uniform random distribution. A routing tree is constructed by each node choosing from its neighbors the node closest to the sink as its next hop. We used the power consumption values as shown in Table 2 and Table 4 represents other related parameters and corresponding values. Most of the parameters are based on S-MAC in ns-2. The SYNC period for all the protocols are set to 55.2 ms. All the period lengths are fixed based on the PHY and MAC layer parameters except the NTP which depends upon the number of delay-intolerant flows.

In our simulations, we exclude the synchronization traffic and assume all the nodes in the network have already been synchronized, but nodes still wake up at the beginning of SP after each synchronization interval (SYNC interval) and listen to the medium. In this study, we compare MQ-MAC with two protocols: PQ-MAC and S-MAC. PQ-MAC is chosen as it is one of the recent QoS-aware duty cycle MAC protocols and we choose S-MAC since it is the most well-known and mostly compared protocol with other duty-cycle schemes. Each simulation has been performed for 1,000 seconds, and we averaged the values obtained for 30 random runs. In all the simulations, the sources start data generation after 100 s from the beginning of the simulation.

### 5.2. Performance Metrics

In this study, we used the following metrics to evaluate MQ-MAC.

*Energy Consumption.* The total energy consumption, *E*, is calculated as

(18) $$E=\sum_{i=1}^{n}P_{l}T_{l}^{i}+P_{t}T_{t}^{i}+P_{r}T_{r}^{i}+P_{p}T_{p}^{i}+P_{s}T_{s}^{i}$$

where *n* is the number of deployed nodes. The average value of *E* is taken after 30 simulation runs.

*End-to-End Latency.* End-to-End latency of a packet is measured as the time difference between the packet generation time and the time when it is received by the sink. Delays experienced by distinct data packets are averaged over the total number of distinct packets received by the sink.

*Reliability.* It is the ratio of the total number of unique packets received by the sink (for delay-intolerant traffic, the number of packets received by the sink within delay deadline is considered) to the total number of packets generated by the source nodes.

### 5.3. Simulation Results

This section presents the results obtained through evaluating MQ-MAC using the metrics stated above. While evaluating end-to-end latency and reliability, we consider broadcast traffic along with two traffic classes. As a delay-intolerant traffic, we choose class 0, and class 2 traffic is chosen as a member of delay-tolerant traffic. We exclude both class 1 and class 3 traffic since both the traffic classes share the same periods with the class 0 and class 2 traffic respectively and hence would have similar behavior. For PQ-MAC, the priority level for the traffic classes is assigned as the following order: class 0 > class 2 > Broadcast. We randomize the initial data generation time of the sources to avoid the synchronized periodic report of the sensors. The sink generate broadcast traffic at a fixed rate of 1 packet per 50 s and for broadcast traffic the E2E latency is measured as the time it takes for a node in the last level of the routing tree to receive a given broadcast packet.

However, during the evaluation of energy consumption, we considered all of the traffic classes and in that case, we assign the following priority order of the traffic classes for PQ-MAC: Class 0 > Class 1 > Class 2 > Class 3, broadcast. The other settings are kept similar as stated above.

In all the evaluations of the performance metrics, we showed the impact of data generation interval, number of sources and delay deadline. In each of the cases, the participating source nodes are equally distributed to the existing number of traffic flows. For example, if two traffic classes are used in a network with eight sources, then, four sources generate one class of traffic while the other four generate the another traffic class. While showing the impact of data generation interval, eight nodes at the margin are chosen as source nodes. The delay deadline for class 0 traffic is set as 4 s and hence, we obtain the *T_o_* for MQ-MAC as 2.14 s. While showing the impact of number of sources, we fixed the data generation interval for all the sources as 10 seconds and vary the number of sources. The delay deadline for class 0 traffic set to 4 s. At the time of exhibiting the impact of delay deadline, we fixed the number of sources as eight. The data generation interval is also set to 10 s and the delay deadline of class 0 traffic is varied up to 6 s.

#### 5.3.1. End-to-End (E2E) Latency

We evaluate the average E2E latency observing the impact of data generation interval, number of source nodes and delay deadline of the traffic.

**Figure 7.**
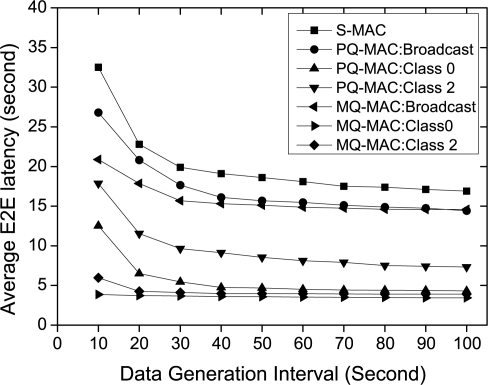
Average E2E latency varying data generation interval.

*Impact of data generation interval.* Figure 7 shows the effect of data generation interval on the average E2E latency. As shown in this figure, the average E2E latency decreases as the traffic load decreases, since the delay incurred due to contention and collision reduces. S-MAC has the highest average E2E latency irrespective of the traffic class (no traffic differentiation), due to the long per-hop delay bounded by the cycle length. The contention and collision delay for S-MAC is also severe, since all types of flows contend simultaneously during the listen period. Being a low priority, broadcast traffic for PQ-MAC also incurs higher delay compared to other traffic classes, although it is lower than S-MAC due to less collision probability. In this case, MQ-MAC has the lowest contention probability and thus the minimum delay for broadcast traffic, because of the allocation of distinct broadcast period. Due to the doubling scheme of PQ-MAC which is based on the priority level, Class 0 traffic has lower delay than Class 2 traffic. However, MQ-MAC has the lowest delay for both Class 0 and Class 2 traffic compared to PQ-MAC, since Class 0 traffic is transmitted using delay-efficient and collision free ASW approach, also the lost packet of Class 2 traffic is retransmitted in the same *T_o_* using this approach. Moreover, contention probability is less for Class 2 traffic due to the separate DTP period.

**Figure 8.**
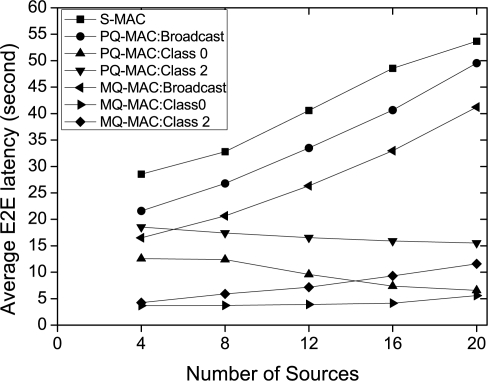
Average E2E latency varying number of sources.

*Impact of number of sources.* Figure 8 exhibits the impact of number of sources on the E2E latency. As the figure shows, the average E2E latency increases as the number of increase almost for each of the cases due to the collision delay. However, PQ-MAC:Class 0 and PQ-MAC: Class 2 shows the reverse trend in this case. The reason is that, in PQ-MAC a node doubles its listen time based on the number of different priority levels of traffic it is transmitting, and this number increases at the forwarding nodes while the number of sources increase which in turn reduces the E2E delay. This also reveals that, doubling scheme of PQ-MAC only works well to reduce the E2E latency for the high priority traffic, while a forwarding node has more diverse packets and thus the packet criticality itself does not suffice to have the lower E2E delay. In contrast, since, MQ-MAC treats each traffic independently according to its QoS requirement, the E2E latency is much lower for Class 0 traffic. However, after a certain traffic load, the delay of MQ-MAC: Class 0 and MQ-MAC: Class 2 increases not for the collision but for the length of NTP and RP which can not accommodate all the relevant packets from the upstream senders in a single wake-up.

**Figure 9.**
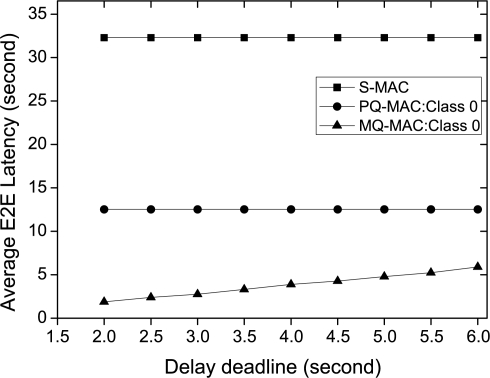
Average E2E latency varying delay deadline.

*Impact of delay deadline.* The impact of delay deadline on average E2E latency for Class 0 packet is illustrated in Figure 9 (we exclude Class 2 traffic as it has no delay deadline). As it shows, for a fixed data generation interval and number of sources, delay deadline for Class 0 traffic has no impact on the average E2E latency for S-MAC and PQ-MAC and in this fixed setting both the protocols fail to meet the delay deadline. However, the average E2E latency increases proportionally with the increase of delay deadline for MQ-MAC as *T_o_* increases and in each of the cases, MQ-MAC successfully meets the delay deadline for Class 0 traffic.

#### 5.3.2. Reliability

In this study, the reliability of diverse packets is evaluated varying the data generation interval and number of sources.

**Figure 10.**
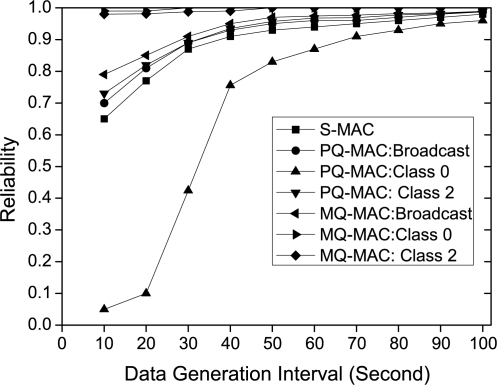
Reliability for different data generation interval.

*Impact of data generation interval.* Figure 10 exhibits the reliability for loss intolerant traffic classes varying the data generation interval. As it shows, the reliability increases for all the cases as the traffic load decreases. Since, S-MAC does not differentiate among traffic classes, thus for S-MAC, we make no distinction while evaluating reliability for different classes for S-MAC only, the reliability for delay intolerant traffic is measured without considering the delay deadline. Considering this, the reliability for S-MAC is the lowest (0.65) at high traffic even compared with the reliability of lowest priority broadcast packet of both PQ-MAC (0.7) and MQ-MAC (0.79). In spite of prioritized traffic handling, PQ-MAC fails to provide higher reliability for Class 0 traffic during the high traffic situation, since most of the packets fail to meet the delay requirements. However, during low traffic rate, the reliability increases to a great extent. PQ-MAC also fails to achieve the higher reliability for Class 2 traffic in high traffic situation. In contrast, in all traffic situation, the loss intolerant traffic classes (Class 0 and Class 2) achieve almost 100% reliability for MQ-MAC.

**Figure 11.**
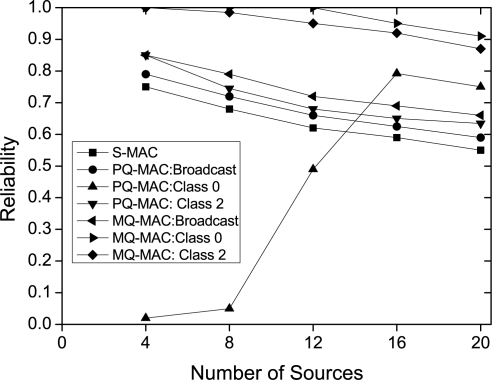
Reliability for different number of sources.

*Impact of number of sources.* Figure 11 illustrates the impact of number of sources on reliability. As depicted in this figure, the reliability decreases with the increase in traffic load in almost all the protocols. The exception is the PQ-MAC: Class 0 traffic in which the reliability increases as the number of sources increase up to certain level. The reason is the lower delay achieved while the number of sources increase as explained earlier. However, the reliability again tends to decrease if traffic load increases much due to collision losses. In contrast, MQ-MAC obtained higher reliability for both Class 0 and Class 2 traffic, also for broadcast traffic compared with the other protocols.

**Figure 12.**
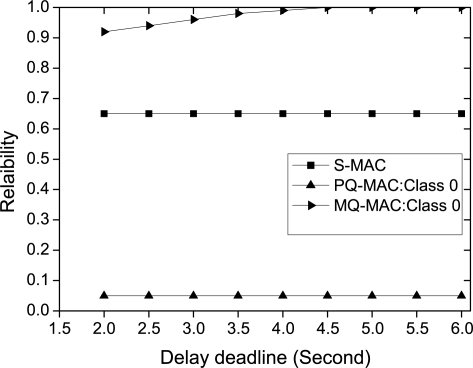
Reliability for different delay requirements.

*Impact of delay deadline.* The impact of delay deadline of Class 0 traffic on the reliability is shown in Figure 12. As the figure exhibits, S-MAC and PQ-MAC has no impact on the reliability with the change of delay deadline since, the operational cycle length of those protocols are always fixed. However, increase in the delay deadline also increases the reliability for the Class 0 traffic in MQ-MAC, due to the dependency of the operational cycle length on the delay deadline. In this case, the sleep time increases which in turn lengthen the NTP and RP duration. Thus, during high traffic, more packets can be transmitted within a single *T_o_* which increases the probability of meeting the delay deadline and hence increases reliability.

#### 5.3.3. Energy Consumption

In this study, we evaluate the average energy consumption of the nodes in the network for different data generation interval, number of sources and delay deadline.

**Figure 13.**
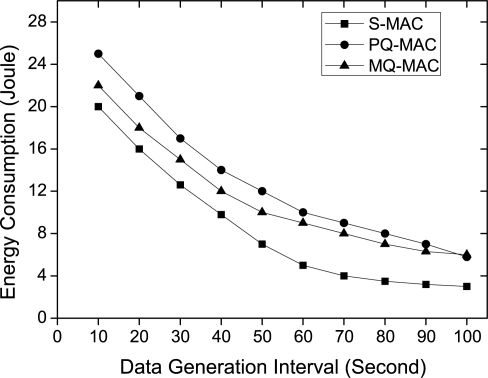
Energy consumption varying data generation interval.

*Impact of data generation interval.* The energy usage varying data generation interval is shown in Figure 13. During high traffic rate, energy depletion is much higher than that of low traffic rate for all the protocols. Among the protocols, nodes in S-MAC consume the least energy due to its least number of wake up in an operational cycle. Due to the doubling listen scheme, PQ-MAC has the highest energy consumption. On the other hand, the energy consumption of MQ-MAC lies in between both the protocols.

**Figure 14.**
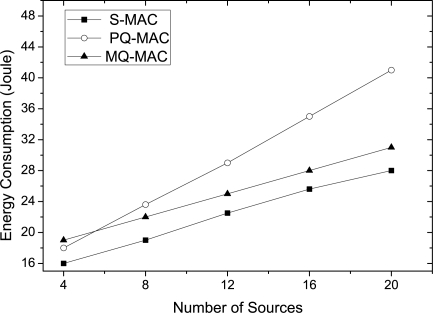
Energy consumption varying number of sources.

*Impact of number of sources.* Figure 14 delineates the effect of number of sources on the energy consumption. As it shows, the energy consumption increases significantly with the growing source number for all the protocols. Particularly, a sharp increase in energy consumption is observed for the nodes in PQ-MAC with higher number of sources. The reason is that, more nodes employ doubling scheme while the number of sources is higher. Although the listen period of PQ-MAC is shorter than MQ-MAC, more state transition occurs for the nodes in PQ-MAC due to the doubling of listen time and this state transition consumes the same energy as the transmission energy. In contrast, nodes in MQ-MAC wake up maximum thrice in an operational cycle (one is in active period, and the other two is in sleep period for transmission and reception) which causes lower energy consumption than PQ-MAC when the number of sources is higher. However, for a very lower number of sources, nodes in PQ-MAC consumes lower energy than that of MQ-MAC.

**Figure 15.**
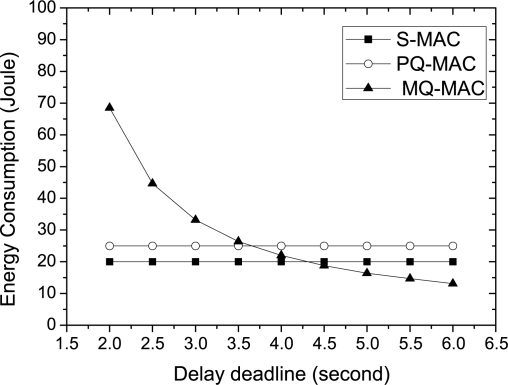
Energy consumption varying delay deadline.

*Impact of delay deadline.* The impact of delay deadline on energy consumption is depicted in Figure 15. As the figure shows, the energy consumption for the nodes in MQ-MAC decreases sharply with the longer delay deadline, since, the sleep time increases as the deadline increases. However, this parameter has no effect on the energy consumption in other protocols.

## 6. Concluding Remarks

This paper presents MQ-MAC, a novel multi-constrained QoS-aware duty cycle MAC for heterogeneous traffic in WSNs. With a classification of traffic based on their multi-constrained QoS demands, MQ-MAC exploits both synchronous and TDMA based asynchronous wake-up scheduling to guarantee both the delay and reliability while achieving energy efficiency.

Through extensive simulation using ns-2, we compared the performance of MQ-MAC with a QoS-aware (PQ-MAC) and a synchronous MAC (S-MAC) protocols. It is evident from the results that, MQ-MAC successfully meets the delay and reliability requirements of diverse traffic when compared to both PQ-MAC and S-MAC. MQ-MAC also achieves higher energy conservation when compared to PQ-MAC, although S-MAC achieves the highest energy consumption than both the QoS-aware duty-cycle MAC protocols with the sacrifice of providing QoS. In summary, with a bit tradeoff in energy consumption, MQ-MAC guarantees the multi-constrained QoS for heterogeneous traffic exist in WSNs.

## Figures and Tables

**Table 1.** Addressed QoS metrics and differentiated service support by the existing duty cycle MAC protocols.

Metric	Differentiated Service	No Differentiated Service
Delay	PQ-MAC [11] QoS-MAC [12]	DMAC [13],R-MAC [5], DW-MAC [14]
Reliability	x	TRAMA [16]

**Table 2.** Typical power values used in Mica2 radio (CC1000).

Symbol	Meaning	Value

*P_l_*	Power in Listening	22.2 mW
*P_t_*	Power in Transmitting	31.2 mW
*P_r_*	Power in Receiving	22.2 mW
*P_p_*	Power in Polling	7.4 mW
*P_s_*	Power in Sleeping	3*μ* W

**Table 3.** Parameters of MQ-MAC in energy model.

Symbol	Meaning	Symbol	Meaning

*R_syn_*	SYNC packet rate	*L_syn_*	SYNC packet size
*R_br_*	Broadcast packet rate	*L_br_*	Broadcast packet size
*R_dtp_*	DT packet rate	*L_lp_*	Wakeup prelude size
$R_{\mathrm{dtp}}^{o}$	Originating rate of DT packet	*L_dtp_*	DT packet size
$R_{\mathrm{dtp}}^{f}$	Forwarding rate of DT transit packets	*L_dip_*	DI packet size
*R_dip_*	DI packet rate	*L_b_*	Beacon packet size
$R_{\mathrm{dip}}^{o}$	Originating rate of DI packet	*T_bo_*	Average backoff period
$R_{\mathrm{dip}}^{f}$	Forwarding rate of DI transit packets	$T_{w}^{\mathrm{br}}$	Waiting time for broadcast packet
$R_{\mathrm{rp}}^{t}$	Transmission rate of retransmitted packets	*N*	Number of neighbors
$R_{\mathrm{rp}}^{r}$	Receiving rate of retransmitted packets		

**Table 4.** Parameters and their values used in the simulation.

Parameter	Value	Parameter	Value	Parameter	Value

Bandwidth	20 kbps	Beacon length	6–8 bytes	*SP*	55.2 ms
SIFS	5 ms	Payload length	50 bytes	*BP*	110.8 ms
DIFS	10 ms	Wakeup-prelude length	1 byte	*DTP*	117ms
Slot time	1 ms	RTS/CTS/ACK length	10 bytes	*RP*	85 ms
Tx Range	250 m	Contention window	64 ms	*SY NC*interval	5 min
Sensing range	550 m	Size of each queue	10 packets	*DataTime_SMAC_*	104 ms
PHY Header	6 bytes	Retry Limit	1	*DataTime_PQMAC_*	165 ms
ChannelEncodingRatio	2	CCA check delay	328*μ*s	*SleepTime*	1,500 ms
